# Development of a clinical trial knowledge management application for community oncology

**DOI:** 10.1093/jamiaopen/ooag140

**Published:** 2026-07-22

**Authors:** Elizabeth O’Brien, Aanshi Thumar, Melanie Manning, Krishna Gunturu, Peter Yu, Tony K W Hung

**Affiliations:** Cancer Institute, Hartford HealthCare, Hartford, CT 06106, United States; Frank H. Netter M.D. School of Medicine, Quinnipiac University, North Haven, CT 06518, United States; Cancer Institute, Hartford HealthCare, Hartford, CT 06106, United States; Cancer Institute, Hartford HealthCare, Hartford, CT 06106, United States; Cancer Institute, Hartford HealthCare, Hartford, CT 06106, United States; Cancer Institute, Hartford HealthCare, Hartford, CT 06106, United States; Cancer Institute, Hartford HealthCare, Hartford, CT 06106, United States; TeamX Health, Los Angeles, CA 91405, United States

**Keywords:** artificial intelligence, clinical trials, accrual, LookUpTrials, user engagement, app development, quality improvement, cancer care engagement

## Abstract

**Introduction:**

Participation in cancer clinical trials is low in community oncology settings, partly because institution-specific trial information is fragmented. We evaluated feasibility of embedding curated trial content in an AI-enabled knowledge management application.

**Materials and Methods:**

At a regional community oncology network, coordinators and disease teams compiled actively recruiting trials. Core elements (title, conditions, biomarkers, stage/line, and recruiting status) were structured for point-of-care display and uploaded. AI-assisted extraction generated protocol summaries and eligibility elements, which underwent systematic human validation.

**Results:**

Fifty-three trials across 10 disease groups were embedded and validated; 91% were recruiting. Trials covered 28 cancer types; 30% were biomarker-specific and most enrolled advanced/metastatic disease. Initial configuration took 2-4 weeks per disease group using existing personnel, without added staffing or electronic health record (EHR) build.

**Discussion:**

Embedding institution-specific trial content within an AI-enabled knowledge application is feasible in community oncology using existing clinical and research infrastructure, establishing a prerequisite for future usability and implementation studies.

## Introduction/background

Clinical trials are the cornerstone of therapeutic advances in healthcare. In oncology—one of the most active areas of clinical research—more than 19 000 clinical trials are actively recruiting in the United States.^1–4^ As therapeutic options become increasingly targeted, thousands of new trials emerge annually, offering patients expanded access to investigational treatments.^5^ However, connecting patients to appropriate trials remains a persistent challenge, particularly within community oncology. Barriers such as complex eligibility criteria, frequent updates to trial availability and protocols, and limited institutional infrastructure contribute to low enrollment rates.^6^^,^^7^ Community oncology programs report a 4.1% participation rate in cancer treatment trials, compared with 21.6% at National Cancer Institute (NCI) designated Comprehensive Cancer Centers and 5.4% at academic (non-NCI) centers.^8^

Healthcare providers are uniquely positioned to support trial enrollment, yet studies indicate that many clinicians lack the time and resources to remain current with the evolving trial landscape.^9–11^ In practice, trial information is often scattered across portals, spreadsheets, and email communications, making it difficult for providers to surface relevant trial options during time-constrained visits. Because patients most often learn about clinical trials directly from their providers, this fragmentation can result in promising trials being overlooked or not discussed in time.^12^ For providers, clear trial information, consistent engagement, and institutional-specific points of contact are critical to supporting trial discussions and sustaining enrollment.^13–18^

To address these barriers, we developed a clinical trial knowledge management platform tailored to the workflows of day-to-day oncology providers.^19–21^ Unlike automated trial-matching tools that focus on identifying candidate studies based on patient characteristics, a clinical trial knowledge management platform is designed to support the “last-mile” execution of trials once a study has been identified. Specifically, it equips clinicians with institution-specific, point-of-care knowledge needed to contextualize eligibility criteria, understand trial logistics, identify appropriate contacts, and engage in informed discussions with patients. The platform is built around 3 core functions: (1) providing real-time, institutional-specific trial information; (2) enabling bidirectional knowledge exchange between clinicians and trial teams; and (3) streamlining trial coordination and referrals toward enrollment.

Following successful deployment of this platform at a National Comprehensive Cancer Center, where it supported 138 oncology trials and maintained user engagement for over 20 months,^20^ this study evaluates the feasibility of developing and validating institution-specific clinical trial content within a community oncology network.

## Methods

### Study setting

This project was conducted at the Hartford HealthCare Cancer Institute (HHCI), a regional network of 7 hospitals and 16 outpatient oncology clinics across Connecticut. The network includes 40 medical oncologists and 14 advanced practice providers who care for more than 9,000 newly diagnosed cancer patients annually, in addition to a large cohort of patients receiving ongoing cancer care.

### Platform development and data curation

Three independent research coordinators collaborated with disease-site oncology teams to compile an inventory of actively recruiting trials. Foundational data fields—trial title, disease area, biomarkers, stage and line of therapy, participating sites, and recruiting status—were structured and entered into the platform. Protocol summaries and eligibility elements were extracted using an embedded retrieval-augmented large language model,^21^ and subsequently reviewed and validated by research coordinators to ensure accuracy, completeness, and institutional relevance. Cancer center leadership, oncologists, and trial coordinators further refined content to optimize clarity for point-of-care decision making.

### Workflow integration and administrative structure

The initial implementation phase required approximately 2 to 4 weeks, after which disease-site study teams assumed administrative ownership of their trial content. Ongoing responsibilities included managing trial availability, updating recruitment status, validating protocol information, and responding to provider inquiries. Study teams set the status of their trials to open (recruiting), open (not recruiting), or closed and were responsible for adding new trials. Research coordinators were designated to support real-time updates and serve as workflow liaisons as needed, with the intent of maintaining engagement and facilitating timely referrals in future phases. The platform did not require EHR integration. Trial content creation and maintenance were incorporated into existing research workflows and personnel responsibilities to minimize operational burden.

### Feasibility assessment

Feasibility was assessed by evaluating whether clinical trial content could be systematically compiled and validated within the platform across the HHCI community oncology network. Descriptive statistics characterized the scope and distribution of trials uploaded, including cancer type, department, biomarker specificity, and stage of disease. Implementation metrics such as the number of recruiting trials represented and coverage across oncology service lines served as indicators of feasibility and breadth of deployment.

## Results

A total of 53 clinical trials across 10 oncology disease groups were systematically compiled and validated into the platform. At the time of reporting, 48 trials (91%) were actively recruiting. Trials represented 28 cancer conditions, with the highest representation in breast (25%), gastrointestinal (13%), and non–small cell lung cancer (11%). One-third of trials (30%) included biomarker-specific eligibility criteria, and most targeted advanced (38%), metastatic (28%), or recurrent (6%) disease.

Trial content was structured using standardized data elements aligned with existing research workflows, resulting in consistent representation across oncology service lines. Three primary administrative roles were defined during content development: research coordinators (initial configuration and support), oncologists (content review and clinical validation), and clinical trial staff (content maintenance). Following initial configuration, disease-site teams assumed responsibility for maintaining trial content, including recruitment-status updates and responses to provider inquiries.

**Figure 1.**
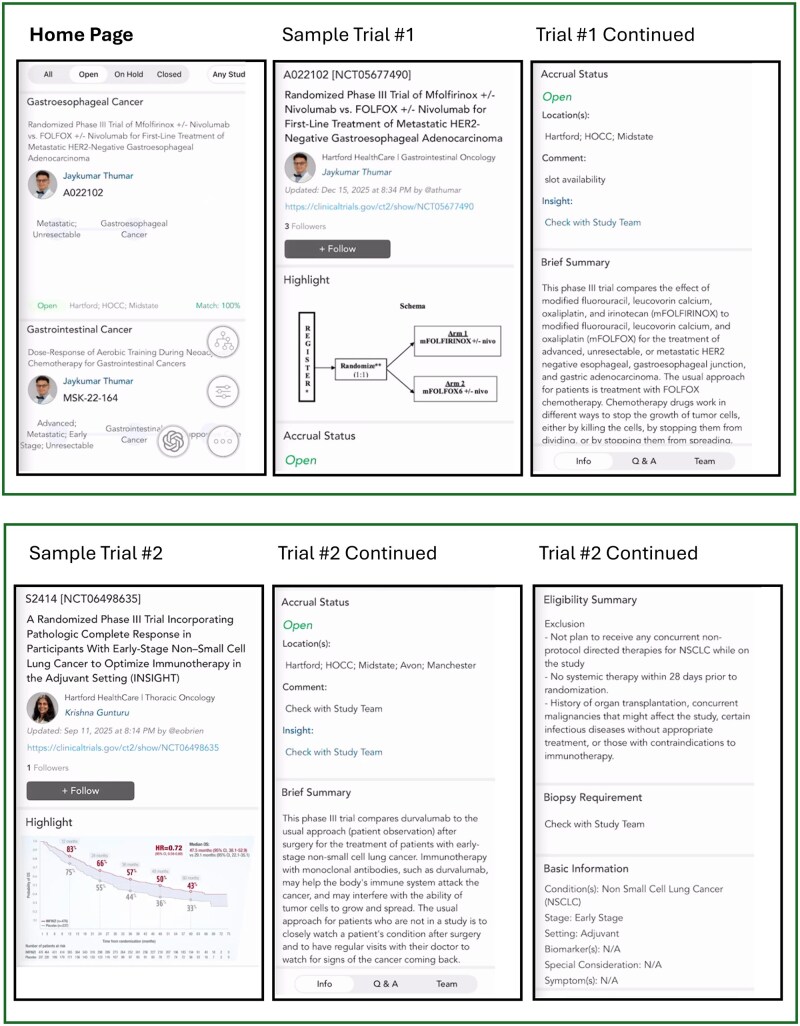
Clinical trial knowledge management application: user interface. Representative screenshots of the application demonstrate how institution-specific clinical trial information is presented to clinicians. The home page displays available clinical trials by disease group and recruiting status. Selecting a trial provides detailed study information, including trial design, eligibility criteria, recruiting status, participating sites, study contacts, protocol summaries, and key trial highlights. Trial content is intended to support point-of-care clinical trial discussions, referrals, and coordination between providers and research teams. Example trials are shown for illustrative purposes. *Brief Summary and Eligibility Summary pages are cut off for brevity. Full information is available on application

Initial platform configuration required limited incremental resources beyond existing clinical research operations. Configuration was completed over a 2- to 4-week period per disease group, averaging approximately 6-8 hours of effort per week by a single team member, and relied on existing personnel roles without the need for additional staffing, dedicated informatics support, or new electronic health record builds. The platform was designed such that ongoing trial content updates could be incorporated into existing workflows. See Figure 1 for current user interface. Summary trial characteristics are presented in Table 1.

**Table 1.** Clinical trial knowledge management application: clinical trial characteristics.

Clinical trial characteristics	Overall, *N* (%)
**All trials**	53
** Open (Recruiting)**	48 (91%)
** Open (Not Recruiting)**	5 (9%)
** Closed**	0 (0%)
**Oncology services**	
** Breast**	8 (15%)
** Gastrointestinal**	7 (13%)
** General**	9 (17%)
** Genitourinary**	6 (11%)
** Gynecologic**	7 (13%)
** Head and neck**	1 (2%)
** Hematologic**	2 (4%)
** Melanoma**	3 (6%)
** Neurologic**	2 (4%)
** Thoracic**	8 (15%)
**Conditions** ^*^	
** Bladder cancer**	4 (8%)
** Breast cancer**	13 (25%)
** Cervical/vaginal cancer**	1 (2%)
** Chronic myelomonocytic leukemia**	1 (2%)
** Colon cancer**	1 (2%)
** Endometrial cancer**	1 (2%)
** Fallopian tube cancer**	3 (6%)
** Gastroesophageal cancer**	1 (2%)
** Gastrointestinal cancer**	7 (13%)
** Genitourinary cancer**	1 (2%)
** Glioma**	2 (4%)
** Gynecologic (general) cancer**	2 (4%)
** Head and neck cancer**	1 (2%)
** Hepatocellular cancer**	1 (2%)
** Lymphoma**	0 (0%)
** Melanoma**	3 (6%)
** Multiple myeloma**	1 (2%)
** Myelodysplastic syndrome**	1 (2%)
** Non-small cell lung cancer**	6 (11%)
** Neuro-oncology cancer**	1 (2%)
** Ovarian cancer**	3 (6%)
** Pancreatic cancer**	2 (4%)
** Peritoneal cancer**	3 (6%)
** Prostate cancer**	4 (8%)
** Rectal cancer**	1 (2%)
** Small cell lung cancer**	1 (2%)
** Thoracic (general) cancer**	4 (8%)
** Urothelial cancer**	1 (2%)
**Biomarkers**	
** No**	37 (70%)
** Yes**	16 (30%)
**Clinical settings** ^†^	
** Advanced**	20 (38%)
** Early stage**	4 (8%)
** Locally advanced**	12 (23%)
** Metastatic**	15 (28%)
** Muscle invasive**	2 (4%)
** Newly diagnosed**	1 (2%)
** Non-muscle invasive**	1 (2%)
** Preventative**	1 (2%)
** Recurrent**	3 (6%)
** Supportive care**	5 (9%)
** Unresectable**	1 (2%)

* Trial may include more than one condition.

† Trial may include more than one clinical setting.

## Discussion

In this study, we demonstrated the feasibility of developing and validating institution-specific content within a clinical trial knowledge management application across a regional community oncology network. Using standardized data elements, 53 trials spanning 10 oncology disease groups were systematically compiled, structured, and validated. Content development was supported by existing clinical and research infrastructure, without the need for additional personnel or informatics resources.

A key finding is that trial content development and validation can occur outside the EHR while remaining aligned with established clinical and research workflows. Use of an external knowledge management application enabled structured content configuration without competing for EHR development capacity. AI-assisted abstraction accelerated initial extraction of protocol summaries and eligibility elements; however, human review was necessary to ensure accuracy and institutional relevance, reinforcing the importance of a human-in-the-loop approach for trial content development.

This work adds to the growing literature on informatics tools supporting clinical trial operations. Prior efforts have focused largely on trial registries and automated patient-trial matching tools, which emphasize trial discovery and eligibility determination.^22–27^ In contrast, a clinical trial knowledge management platform focuses on surfacing the local trial information required once a study has been identified–such as practical eligibility nuances, recruitment status, and operational contacts–that are critical to support informed discussion of trials and not routinely captured in registries. Establishing this type of institution-specific trial content is particularly relevant in community oncology settings, where limited infrastructure and fragmented information have historically contributed to disparities in access to clinical research.^28^^,^^29^

Several study limitations warrant consideration. This analysis was limited to feasibility of content development and validation and did not assess application usability, workflow adoption, provider behavior, referral activity, or trial accrual. The findings reflect experience within a single community oncology network with established research infrastructure and may not generalize to settings with fewer resources.

Despite these limitations, this work demonstrates the feasibility of developing and validating institution-specific clinical trial content within a knowledge management application for the community oncology setting. Establishing this content layer is a prerequisite for subsequent evaluations of the application usability, implementation barriers and facilitators, and its downstream effects on clinical trial referral and enrollment. Ongoing studies are underway to address these dimensions as part of a broader research program. Future research will also explore how such platforms may be optimized and extended to additional stakeholders, including patients and trial sponsors, as part of a more coordinated clinical research ecosystem.

## Data Availability

The data that support the findings of this study are available from the corresponding author upon reasonable request.
